# Comparing natural language processing representations of coded disease sequences for prediction in electronic health records

**DOI:** 10.1093/jamia/ocae091

**Published:** 2024-05-08

**Authors:** Thomas Beaney, Sneha Jha, Asem Alaa, Alexander Smith, Jonathan Clarke, Thomas Woodcock, Azeem Majeed, Paul Aylin, Mauricio Barahona

**Affiliations:** Department of Primary Care and Public Health, Imperial College London, London, W12 0BZ, United Kingdom; Department of Mathematics, Centre for Mathematics of Precision Healthcare, Imperial College London, London, SW7 2AZ, United Kingdom; Department of Mathematics, Centre for Mathematics of Precision Healthcare, Imperial College London, London, SW7 2AZ, United Kingdom; Department of Mathematics, Centre for Mathematics of Precision Healthcare, Imperial College London, London, SW7 2AZ, United Kingdom; Department of Epidemiology and Biostatistics, Imperial College London, London, W2 1PG, United Kingdom; Department of Mathematics, Centre for Mathematics of Precision Healthcare, Imperial College London, London, SW7 2AZ, United Kingdom; Department of Primary Care and Public Health, Imperial College London, London, W12 0BZ, United Kingdom; Department of Primary Care and Public Health, Imperial College London, London, W12 0BZ, United Kingdom; Department of Primary Care and Public Health, Imperial College London, London, W12 0BZ, United Kingdom; Department of Mathematics, Centre for Mathematics of Precision Healthcare, Imperial College London, London, SW7 2AZ, United Kingdom

**Keywords:** Multiple Long Term Conditions, Long-Term Conditions, diseases, representations, prediction, natural language processing

## Abstract

**Objective:**

Natural language processing (NLP) algorithms are increasingly being applied to obtain unsupervised representations of electronic health record (EHR) data, but their comparative performance at predicting clinical endpoints remains unclear. Our objective was to compare the performance of unsupervised representations of sequences of disease codes generated by bag-of-words versus sequence-based NLP algorithms at predicting clinically relevant outcomes.

**Materials and Methods:**

This cohort study used primary care EHRs from 6 286 233 people with Multiple Long-Term Conditions in England. For each patient, an unsupervised vector representation of their time-ordered sequences of diseases was generated using 2 input strategies (212 disease categories versus 9462 diagnostic codes) and different NLP algorithms (Latent Dirichlet Allocation, doc2vec, and 2 transformer models designed for EHRs). We also developed a transformer architecture, named EHR-BERT, incorporating sociodemographic information. We compared the performance of each of these representations (without fine-tuning) as inputs into a logistic classifier to predict 1-year mortality, healthcare use, and new disease diagnosis.

**Results:**

Patient representations generated by sequence-based algorithms performed consistently better than bag-of-words methods in predicting clinical endpoints, with the highest performance for EHR-BERT across all tasks, although the absolute improvement was small. Representations generated using disease categories perform similarly to those using diagnostic codes as inputs, suggesting models can equally manage smaller or larger vocabularies for prediction of these outcomes.

**Discussion and Conclusion:**

Patient representations produced by sequence-based NLP algorithms from sequences of disease codes demonstrate improved predictive content for patient outcomes compared with representations generated by co-occurrence-based algorithms. This suggests transformer models may be useful for generating multi-purpose representations, even without fine-tuning.

## Introduction

Due to population aging and the effective management of many diseases, an increasing number of people are living with Multiple Long-Term Conditions (MLTC), a health state defined as the co-occurrence of 2 or more chronic or Long-Term Conditions (LTCs), and associated with a range of adverse health outcomes.^1^^,^^2^ Understanding the determinants and consequences of MLTC has become a priority for medical research and represents a significant shift from a focus on individual diseases to a focus on understanding the interactions of combinations of LTCs.^2–4^ Electronic health records (EHRs), particularly those from primary care settings, which capture the history of a person’s diseases over time, provide a powerful resource for exploring these interactions, and inform predictive applications that may be used within the EHR in clinical practice. The growing use of EHRs in medical research has promoted the translation of methods which can handle such big data such as those developed in natural language processing (NLP). Applied to the structured data in EHRs, the temporally-ordered sequence of medical codes or diseases in a patient’s record can be viewed as analogous to the sequence of words in a sentence or document.^5^ NLP approaches have previously been used to generate representations of patients based on their sequence of diseases^6–8^; more recent transformer architectures can also be “fine-tuned” to optimize the learned representation for prediction of clinical endpoint, such as the next disease.^5^^,^^9^

In healthcare settings, there are advantages to learning a single unsupervised representation of a patient, which can be used across a range of applications. Although fine-tuning the representations for every outcome of interest may improve the predictive performance for each individual outcome, this will come at cost of its generalizability to other applications. For example, segmentation methods used in healthcare aim to cluster patients for risk stratification, based on measures of similarity derived from demographic and clinical criteria; if 1 representation performs well across a range of outcomes, then only 1 representation and clustering could be trained and implemented, reducing complexity.^10^ However, there is little comparative research of the unsupervised representations of a person’s disease history generated by different NLP methods, and how these compare to standard epidemiological approaches in prediction of clinical outcomes. Furthermore, the optimal input strategies when translating methods designed for natural language to structured healthcare data remain uncertain. When analyzing EHR data, a common approach is for clinical experts to manually group individual clinical codes (for example, those represented by International Classification of Diseases (ICD) or Systematized Nomenclature of Medicine Clinical Terms (SNOMED-CT) ontologies), into disease categories, to reduce the number of inputs into a model. This process is time-consuming, may be subjective, and risks discarding relevant information captured in a more specific code. If use of individual clinical codes alone can perform as well, or better than disease groupings, this might reduce the need for clinically derived categories as inputs for predictive models.

In this study, we compare different patient representations generated from disease code sequences by applying widely used bag-of-words and sequence-based NLP methods: Latent Dirichlet Allocation (LDA), doc2vec, and 2 transformer architectures designed for EHR data: Med-BERT and BEHRT.^5^^,^^9^ We also develop a new transformer architecture EHR-BERT that includes additional sociodemographic information which we hypothesize might improve the predictive content of the disease representations. For each model, we compare as inputs the use of disease categories versus a larger vocabulary of individual clinical codes. We then evaluate the predictive content of these unsupervised patient representations when used as input features to a simple logistic classifier to predict 5 clinically relevant outcomes over 1 year: mortality, emergency department (ED) attendance, emergency hospital admission, attendance with a condition, and new diagnosis of a condition. The obtained embeddings are also compared against a baseline epidemiological approach, where individual diseases are used as input features to the logistic classifier. By focusing on the comparative performance across different predictive tasks, our aim is not to develop a best-performing classifier pipeline as an end-product, but rather, to assess if increasingly complex embedding representations of patient’s diseases can enhance downstream predictive performance and could inform future healthcare applications across a range of healthcare data contexts and tasks.

## Methods

### Data sources

We used the Clinical Practice Research Datalink (CPRD) Aurum, a nationally representative EHR dataset from General Practices (GPs) in England.^11^ Our data extract includes all patients aged 18 years or over, marked as “research acceptable” (a data quality marker defined by CPRD, such as including a valid date of birth and registration date^12^) and registered to a GP practice on January 1, 2015. We included all patients with 2 or more LTCs (defined below) eligible for linkage to secondary care data and those registered to a practice for at least 1 year before January 1, 2015, to ensure sufficient time for data input.^13^ Linkage was performed by NHS Digital; patients not eligible for Hospital Episode Statistics (HES) linkage included those with missing or incorrect data on 1 or more of: National Health Service number, sex, date of birth, or postcode.^14^

Cleaning rules for demographic data including age, gender, and ethnicity are given in the Supplementary Information, p. 2-3. As a marker of socioeconomic deprivation, data were linked to deciles of the 2019 Index of Multiple Deprivation (IMD) of a patient’s geographical area of residence, a composite measure capturing a broad range of socioeconomic factors.^15^ Missing sociodemographic data (see Table 2) were treated as missing indicator variables in all analyses. Secondary care data was sourced from HES and death registrations from the Office for National Statistics, both linked to the Aurum dataset by CPRD.^16^ As CPRD Aurum data also includes a marker of mortality, any differences in the dates between sources were reconciled using a modification of the algorithm recommended by Delmestri and Prieto-Alhambra (2020) (see Supplementary Information, p. 3).^17^

**Table 2.** Characteristics of the patient cohort (*N* = 6 286 233).

Patient characteristic	Total	Percent
**Age (years)**		
Mean (standard deviation)	53.8 (18.2)	
18-29	707 208	11.3
30-39	803 366	12.8
40-49	1 108 696	17.6
50-59	1 225 401	19.5
60-69	1 081 293	17.2
70-79	797 941	12.7
80+	562 328	8.9
**Gender**		
Female	3 339 743	53.1
Indeterminate	58	<0.1
Male	2 946 432	46.9
**Ethnicity**		
White	5 419 210	86.2
South Asian	372 336	5.9
Black	220 687	3.5
Other	83 472	1.3
Mixed	64 109	1.0
Missing	126 419	2.0
**IMD decile**		
1 (least deprived)	710 805	11.3
2	652 898	10.4
3	665 105	10.6
4	662 155	10.5
5	594 419	9.5
6	624 994	9.9
7	622 678	9.9
8	598 585	9.5
9	598 129	9.5
10 (most deprived)	552 291	8.8
Missing	5174	0.1
**Total**	**6 286 233**	

### Disease definition and sequence construction

Coded data entered during clinical encounters are stored in CPRD as numeric “Medcodes”, a disease ontology specific to the dataset which maps to both SNOMED and Read codes.^18^ We included 9462 Medcodes representing a group of 212 LTCs based on earlier research in which Medcodes were assigned by clinicians to disease categories.^19–21^ For example, Medcodes representing “Allergic asthma” (Medcode ID: 1483199016) or “Intrinsic asthma” (Medcode ID: 396114013) are grouped under the disease “Asthma”. The mapping is available from https://tbeaney.github.io/MMclustering/.

All retrospective clinical codes for each patient are included in CPRD (ie, before the study start date), where clinical diagnoses may be back-dated to the date of diagnosis (“observation date”) rather than the date of recording (“entry date”). For each patient, we constructed a sequence of diagnostic codes ordered by the observation date representing the full history of Medcodes documented in the EHR from birth up to (but not including) January 1, 2015. Any clinical data with an observation or entry date from January 1, 2015 were excluded. We created 2 separate sequences: the first, “Diseases” sequence using the disease categories (vocabulary size = 212), and the second, “Medcodes” sequence using the individual Medcodes (vocabulary size = 9462). Given our inclusion of patients with 2 or more LTCs, all “Diseases” sequences contained at least 2 different diseases and all “Medcodes” sequences contained at least 2 different Medcodes. For example, a “Diseases” sequence for a patient might read “asthma, rheumatoid arthritis, depression, atrial fibrillation, depression.” In contrast to other studies that apply NLP algorithms directly to text data, our sequences were limited to coded data, and hence we did not need to undertake additional pre-processing steps.

### Patient representation methods

**Figure 1.**
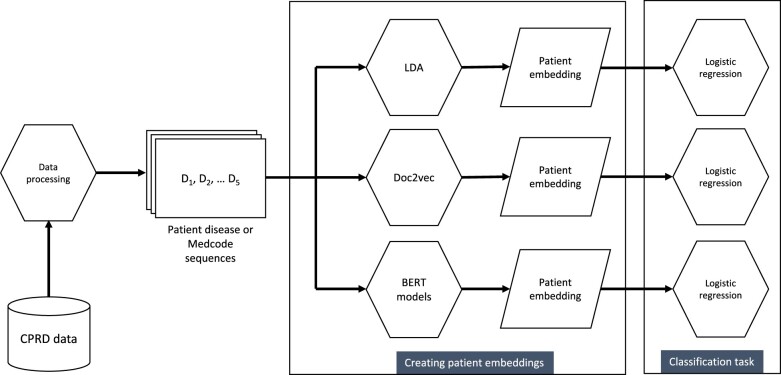
Analysis pipeline for generating patient embeddings and predicting outcomes. Separate embeddings generated using disease and Medcode sequences. BERT models include Med-BERT, BEHRT, and EHR-BEHRT and use additional inputs as described in the methods. Patient embeddings created using data recorded before 2015, classification task using data recorded in 2015.

We applied a range of NLP methods originally designed for deriving document representations (embeddings) from text, but in our case, we apply them to disease (or code) sequences for a patient, thus interpreting the order of diseases in a person as analogues to words in a document. The result is then an embedding that encapsulates the patient disease sequence. For each method, we ran separate models using the disease sequences or Medcode sequences (see Figure 1 for our analysis pipeline).

### Latent Dirichlet allocation

Latent Dirichlet allocation is a co-occurrence-based generative probabilistic model which applies a distribution of topics over each document, assuming topics are drawn from a Dirichlet distribution.^22^ Details of our model set-up and choice of priors are given in the Supplementary Information. To select the optimal number of topics, we divided our data into an 80:20 train-test split and selected the number of topics resulting in the lowest value of the perplexity score on a test set not seen during training (see Supplementary Information, p. 4-6).^23^ Using the optimal number of topics, we then repeated the LDA algorithm on the full dataset to generate topic distributions for the full patient cohort.

### Doc2vec

Doc2vec is an extension of the word2vec algorithms, which directly learns vector representations of text sequences ranging in length from sentences to documents.^24^ Further details of our hyperparameter tuning are given in the Supplementary Information, based on our previous work using word2vec.^21^ We found that the default learning rate and epochs for text produced poor representations of the patient embeddings, when using the smaller vocabulary of diseases and so evaluated the performance of doc2vec models using different learning rates and epochs. Here, we made use of patients with identical sequences in the record, who we would expect to have a similar embedding vector (further details given in the Supplementary Information, p. 7-11). We compared both Distributed Bag of Words (DBOW) and Distributed Memory (DM) algorithms for both disease and Medcode sequences. The DM model learns features by approximating individual words (diseases or Medcodes in our case) using the surrounding context and the DBOW model samples a random word from a patient sequence to approximate the surrounding context. The former approach takes order into consideration, but is more likely to learn similar patterns with smaller lengths of sequence.^24^

### Transformer models

We compared Med-BERT and BEHRT, 2 recent transformer-based architectures designed to use coded EHR data. Med-BERT used as input 82 000 ICD-9 and ICD-10 codes from hospital EHR data for 20 million patients,^5^ whereas BEHRT grouped codes from 1.6 million patients’ primary care EHR data into 301 disease categories, and supplemented training with information on patient age at each code occurrence.^9^ Although the original BERT implementation applied to language data included an additional pre-training step with a next-sentence prediction task, further studies have suggested improvement in downstream language tasks using masked-language-modelling (MLM) alone.^25^ We therefore used MLM alone (as done in BEHRT) and did not conduct the further pre-training step performed in Med-BERT. The MLM approach in a transformer architecture enables the model to learn a deep, bidirectional representation of an entire sequence. The attention mechanism, which is a mainstay of the transformer-based architectures, allows the model to capture longer range of dependencies between the EHR codes, leading to better capturing of context. To better enable direct comparison between models, we made some changes to the default implementations and fixed the minimum sequence length per patient to 2, and the maximum sequence length to 128, which accounted for the full sequence for 97.3% of patients (Table 1). For the remaining 2.7% of patients, sequences longer than 128 were truncated, retaining the most recent 128 codes.

**Table 1.** Model inputs and default parameters for Med-BERT, BEHRT, and EHR-BERT architectures.

	Med-BERT	BEHRT	EHR-BERT
**Model inputs**			
Minimum visits per patient^a^	3	5	2
Maximum sequence length per patient^b^	512	64	128
Input embeddings (in addition to code input)	Visit number ± absolute position	Visit number, age, and segment	Visit number, age, gender, ethnicity, deprivation, calendar year
Use of [CLS] and [SEP] tokens	Not used	[CLS] at start of sequence and [SEP] between each visit	[CLS] at start of sequence and [SEP] at end of sequence
**Model parameters**			
Batch size	32	256	256
Hidden layers	6	6	6
Attention heads	6	12	12
Hidden size	192	288	288
Intermediate size	64	512	512
Learning rate	5e-5	3e-5	3e-5

a In contrast to the original Med-BERT and BEHRT models, we used a minimum sequence length of 2 in all configurations to include all patients with MLTC.

b In contrast to the original Med-BERT and BEHRT models, we used a maximum sequence length of 128 in all configurations.

We also developed a new transformer architecture, which we call EHR-BERT. EHR-BERT adapts and extends the BEHRT model to include additional sociodemographic factors which we found in earlier work to be strongly associated with code frequency in the EHR.^26^ We added embedding layers for gender, ethnicity, IMD decile, and calendar year of the observation (Table 1 and Figure S4). Compared with BEHRT, we removed the segment token, which in the original BERT paper related to inputs of sentence pairs which is not applicable in our case. We also removed the separator ([SEP]) tokens which separate consecutive visits, given most visits contained only 1 code, and visit number should suffice to capture sequential visit information.

The 6 models (3 BERT variants, each with 2 separate model inputs of diseases or Medcodes) were trained on the full study cohort for 100 epochs, as used in BEHRT. To generate patient embeddings, we averaged the second-to-last hidden layer of each of the contextualised disease embeddings in a patient’s sequence, resulting in a vector equal in length to the hidden size.^27^^,^^28^

### Evaluation

We evaluated the performance of the embeddings (created using clinical codes that occurred and were recorded before 2015) in predicting the following binary outcomes that occurred in 2015, ie, using data not seen during the unsupervised training of the embeddings:

1. Mortality (of patients aged ≥60 years on January 1, 2015)
2. Any ED attendance
3. Any emergency hospital admission
4. Any attendance with a coded diagnosis code for:
  - Hypertension
  - Diabetes (Type 1 or Type 2, or unspecified)
  - Depression
5. A new diagnosis of:
  - Hypertension
  - Diabetes (Type 1 or Type 2, or unspecified)
  - Depression

We focused on evaluating outcomes over 1 year, as a timeframe relevant to health services, for example, by identifying patients at higher risk of death, and healthcare utilization to prompt interventions. Given mortality is a relatively rare event across the whole population, we explored mortality in the 60+ year cohort alone, but for other outcomes, did not restrict by age. The 3 diseases were selected based on relatively high frequency in the dataset (Table S2) and also based on feedback from our patient and public involvement panel, which included 3 people with lived experience of having MLTC. Reasons for selecting the 3 conditions included their relative prevalence and impact on health services, perceived importance of early detection for preventive strategies and significance of complications. For each disease, we separately examined “any” attendance with a condition, to “new” (incident) diagnoses—the former indicate active presentations or management for a condition, whereas the latter are likely to be of most interest for early diagnosis and intervention. We excluded 305 142 patients who deregistered during the 12-month period, to ensure equal follow-up time (total population = 5 981 091). For all outcomes except mortality, we also excluded the 85 260 patients who died during the 12-month follow-up (total population = 5 895 831). For prediction of new diagnoses only, we additionally excluded any patient from analysis who already had the disease of interest diagnosed before January 1, 2015. Diseases with an observation or entry date after 2015 were excluded.

As a comparison to standard epidemiological approaches without information on co-occurrence or sequence, we constructed a “Binary disease indicators” embedding, where each of the 212 disease categories was represented as a binary feature (1 = present; 0 = not present), and a “Disease frequency counts” embedding, where each feature represented the number of times each disease appeared in the patient’s record.

To compare the predictive content of the different representations, we used each of them as inputs (features) into a simple logistic regression classifier, and trained separate logistic regression models for each of the embeddings and outcomes described above. Logistic regression models were trained with 4-fold cross-validation, with L2 penalization (regularization strength of 1) and maximum iterations of 100. We experimented with smaller and larger values of the regularization parameter (0.01, 0.1, and 10) but found 1 to be optimal. We included age, gender, ethnicity, and deprivation decile as covariates in all models, categorized as in Table 2. We also compared use of these covariates alone as inputs into the logistic classifier (“Sociodemographics only”). As a sensitivity analysis, we compared model performance using the patient representations alone, without inclusion of covariates.

Models were evaluated on the test sets (2015 data only) using 2 scores: the Receiver Operating Characteristic Area Under the Curve (ROC-AUC) and the Average Precision Score (APS), which is the area under the precision-recall curve and gives a better indication of model performance for predicting the positive class.^29^ In the Supplementary Information, we also report on the sensitivity, specificity, positive predictive value (PPV), and negative predictive value (NPV) for selected embeddings and outcomes, to aid clinical interpretability. As a sensitivity analysis for the Med-BERT, BEHRT, and EHR-BERT models, we compared the performance at 10, 50, and 100 epochs, and selected 100 epochs as the best performing (see Tables S5 and S6).

### Implementation

We used Python version 3.10.9 and Pandas version 2.0.3 for data manipulation and management.^30^^,^^31^ LDA and doc2vec were applied using the gensim library,^32^ and our final selected models took approximately 2 hours and 4 hours, respectively, to run on a single CPU. The BEHRT model was implemented using the authors’ source code available on GitHub (https://github.com/deepmedicine/BEHRT). We implemented Med-BERT and EHR-BERT using the HuggingFace Transformer library.^33^ Med-BERT used the same architecture and hyperparameter settings described by the authors.^5^ All transformer models were run on an NVIDIA Quadro RTX 8000 GPU with 48GB RAM; each model took approximately 10 days to run for 100 epochs.

### Ethics

Data access to CPRD and ethical approval was granted by CPRD’s Research Data Governance Process on April 28, 2022 (Protocol reference: 22_001818) and with linkage to HES data on March 6, 2023 (Protocol reference: 22_002481).

## Results

### Data description

**Figure 2.**
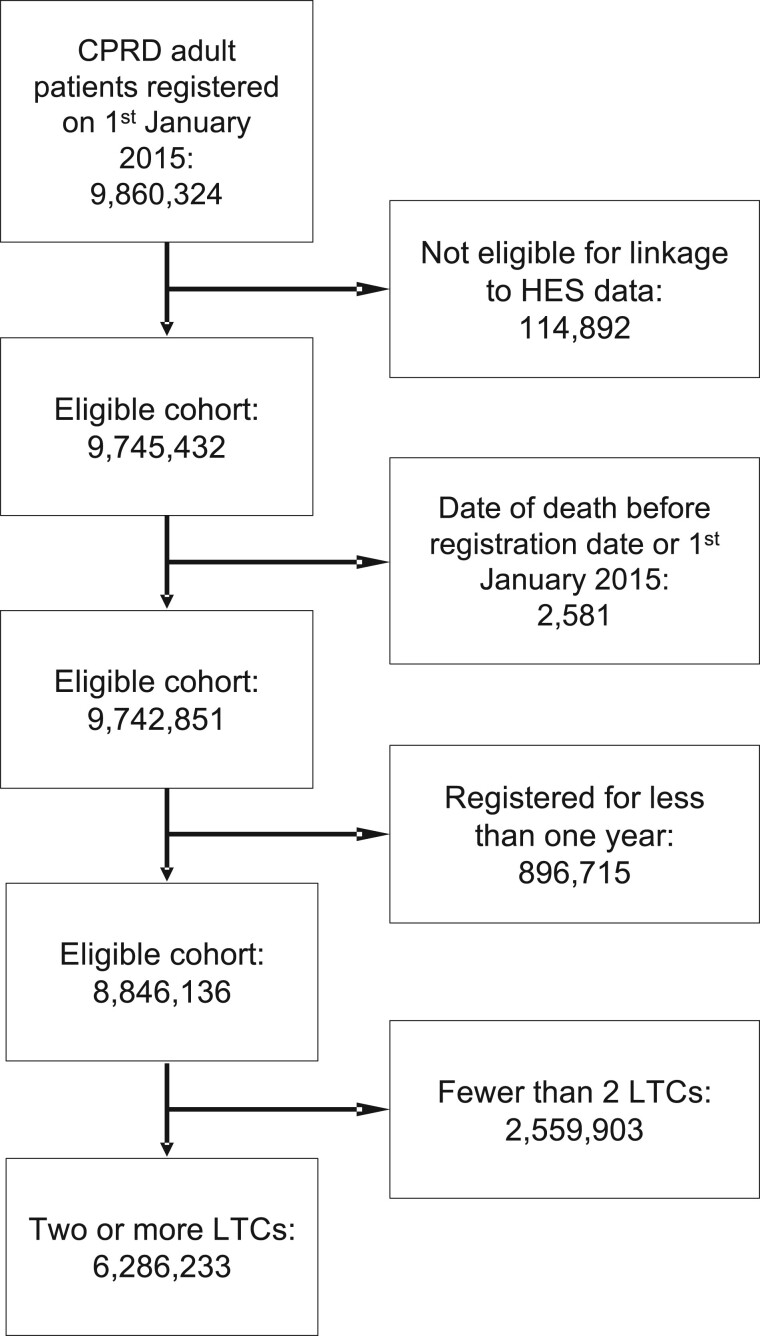
Flow chart of patients included in the study for generating embeddings.

A total of 6 286 233 patients registered to GP practices in CPRD on January 1, 2015 with 2 or more LTCs were included (see Figure 2). Characteristics of the study population are displayed in Table 2. The mean (standard deviation) age of the population was 53.8 (18.2) years, with slightly more females (53.1%) compared to males (46.9%). The majority were of White ethnicity (86.2%), followed by South Asian (5.9%) and Black (3.5%). There was a roughly even spread across the 10 deciles of socioeconomic deprivation as measured by IMD, but with slightly more in the 5 least deprived deciles (52.3%).

### Generating patient representations

We generated vector representations of each patient using different NLP methods, comparing diseases versus Medcodes as inputs (Figure 1). Using LDA, we identified an optimal number of topics of 70 for diseases as inputs and 100 for Medcodes as inputs, based on the lowest perplexity score (see Figures S2 and S3). With doc2vec, we found the DBOW algorithm to perform better than the DM algorithm in assigning patients with the same sequence as similar to each other (Tables S3 and S4) and selected optimal models generating embeddings of size 100 for both disease and Medcode inputs. Finally, we created embeddings using the 3 transformer models, Med-BERT, BEHRT, and EHR-BERT, each trained for 100 epochs.

### Evaluation of embedding performance

We evaluated the predictive content of the patient embeddings generated from each algorithm by using them as inputs (features) for a logistic classifier with the task of predicting clinical outcomes over the subsequent 12 months, ie, using test data that was unseen during training of the embeddings. Event rates for each outcome varied, as displayed in Table 3. We also compared the performance of the classifier using embeddings, to a logistic classifier using as inputs: binary indicators for having a disease (“Binary disease indicators”), count of codes for each disease (“Disease frequency counts”), or sociodemographic covariates alone. The sociodemographic covariates listed in Table 2 were also included in all the classifier models.

**Table 3.** Number of events, denominator populations, and event rates for each outcome during follow-up.

Outcome	Total events	Denominator population	Event rate (%)
60+ mortality	77 349	2 362 981	3.3
Any ED attendance	1 172 555	5 895 831	19.9
Any emergency admission	428 902	5 895 831	7.3
Hypertension—any	619 301	5 895 831	10.5
Diabetes—any	536 320	5 895 831	9.1
Depression—any	227 239	5 895 831	3.9
Hypertension—new diagnosis	63 880	4 402 817	1.5
Diabetes—new diagnosis	42 120	5 336 863	0.8
Depression—new diagnosis	53 203	4 703 263	1.1

**Figure 3.**
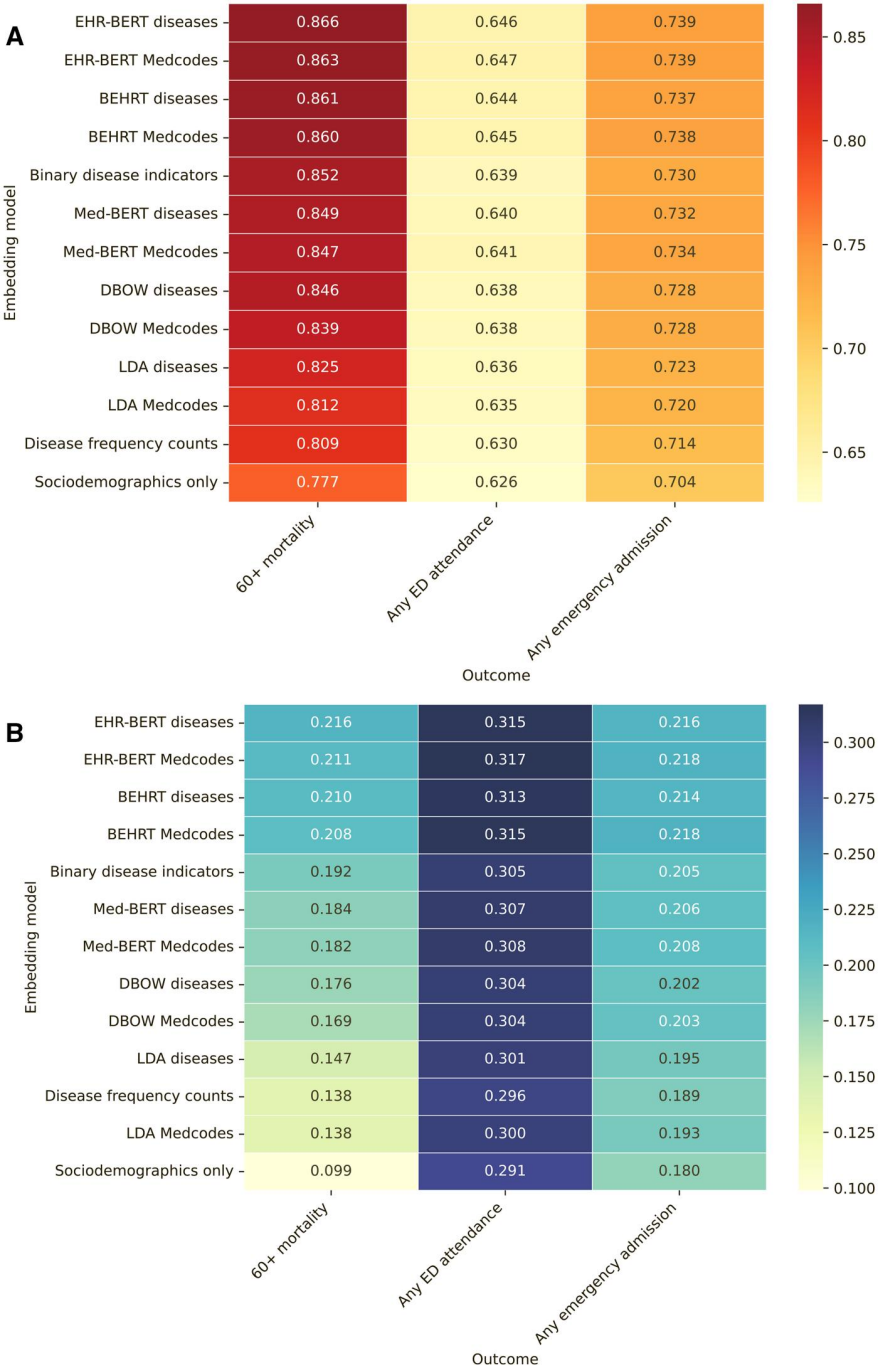
Model ROC-AUC (A) and APS (B) for different embedding models for prediction of mortality in the 60+ age group, emergency department attendances and emergency admissions within 12 months. Embedding models ordered by highest scores for mortality. Abbreviations: APS = average precision score; ROC-AUC = receiver operating characteristic area under the curve.

We first assessed the performance, as measured by AUC and APS, of the patient embeddings generated from each algorithm at predicting mortality (in the 60+ age group), ED attendance, and emergency admissions (Figure 3). The embeddings tended to perform well at predicting mortality, but relatively poorly at predicting any ED attendance. Across all endpoints, embeddings generated by EHR-BERT performed best, followed by those from BEHRT, with use of binary disease indicators performing similarly to Med-BERT. The predictive model using the count of diagnosis codes for each disease (“Disease frequency counts”) had the second lowest performance for all 3 endpoints, ahead only of models using sociodemographic covariates alone.

**Figure 4.**
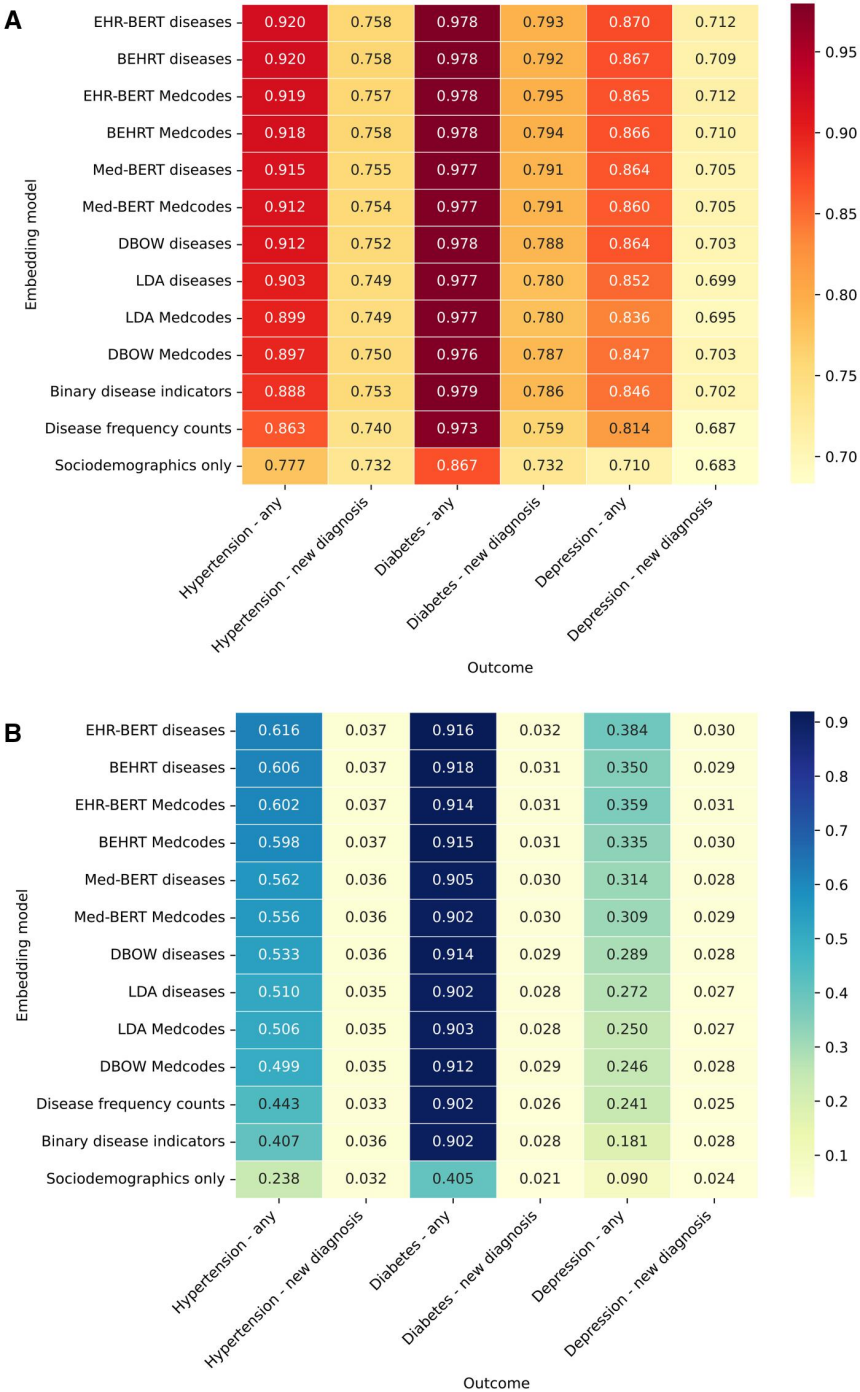
Model ROC-AUC (A) and APS (B) for different embedding models for prediction of any versus new diseases developed within 12 months. Embedding models ordered by highest scores for hypertension (any). Abbreviations: APS = average precision score; ROC-AUC = receiver operating characteristic area under the curve.

We next assessed the performance of the embeddings in predicting any attendance with hypertension, diabetes, or depression over 12 months, versus models predicting new incidences of these diseases amongst patients not already diagnosed (Figure 4). For all diseases, the embeddings were better at predicting any occurrence than predicting newly incident diseases. Embeddings generated by EHR-BERT again performed best across all 3 diseases, followed by those from BEHRT and Med-BERT, and use of binary disease indicators performed comparatively worse. APS scores were consistently low for predicting new diseases.

In the Supplementary Information (pp. 16-18), we provide examples of the AUC curves and metrics of predictive performance under different model sensitivity thresholds, for the EHR-BERT diseases model, compared with the “Sociodemographics only” model, for mortality (Figure S5 and Table S7), ED attendance (Figure S6 and Table S8) and incident diabetes (Figure S7 and Table S9). For example, the PPV for predicting mortality using the EHR-BERT diseases embeddings was 23.7%, 14.1%, and 7.7% for sensitivity thresholds of 40%, 70%, and 90%, respectively, indicating that there were more false positives than true positives, even at low sensitivity thresholds. However, this was substantially better than the “Sociodemographics only” model, with PPVs of 10.7%, 8.6%, and 5.1% at each sensitivity threshold. For ED attendances, there was only a modest improvement of the EHR-BERT diseases embeddings.

A sensitivity analysis running all the classifier models using the embeddings alone as inputs, without addition of the sociodemographic covariates, showed worse overall performance, and similar relative differences with greater absolute differences between the embeddings (Figures S8 and S9). However, for BEHRT, which includes age, and EHR-BERT, which includes sociodemographic variables in the MLM training, addition of sociodemographic covariates to the logistic regression classifier made little difference to the overall performance of the embeddings from these models.

## Discussion

Using a representative cohort of 6 million people with MLTC, we compared different inputs and algorithms to learn representations of patients’ structured disease code patterns as recorded in the primary care EHR and evaluated the predictive content of these representations for a range of clinically relevant health outcomes. There are 3 key findings from our work. First, we directly demonstrated the improvement of NLP representations which incorporate information on disease sequence. There were small additive improvements from Med-BERT to BEHRT to EHR-BERT, of which the latter incorporates additional sociodemographic information in generating embeddings. Although the absolute performance of representations may vary across different healthcare data sources and clinical ontologies, the consistently better predictive content of the representations across all our evaluated outcomes suggests their generalizability to other health settings and tasks. The bag-of-words LDA performed comparatively poorly, and in general were worse than use of simple binary disease indicators often used in health-related applications, which performed relatively well at predicting mortality and healthcare utilization. Second, we found that embeddings generated from EHR-BERT and BEHRT performed better across all tasks, even without fine-tuning, indicating the ability of these unsupervised representations to capture latent information on sequence relevant to a range of health outcomes. Finally, across all embedding algorithms, we found little difference in the predictive performance when using either the large vocabulary of Medcodes, or the smaller vocabulary of clinically categorized diseases.

### Implications for prediction models

While the absolute performance of models for prediction was low for some of the endpoints examined, particularly for ED attendance and new disease diagnoses, our embeddings were generated using only on a small portion of the information recorded in the EHR, namely the coded disease sequences and sociodemographic covariates. However, the EHR is a rich source of additional structured data, including symptoms, examination findings, pathology results, and prescriptions, all of which may aid predictive performance. Although the inclusion of such inputs may not be possible using standard regression techniques, embedding architectures such as transformers offer the ability to incorporate such information and reduce the dimensionality of complex data while also offering improvement in the predictive content compared with co-occurrence methods. As such, transformers including EHR-BERT are promising approaches for predictive applications, and future research should explore inclusion of additional EHR data inputs.

Although representations from transformer models performed best across all tasks, we found that using binary disease indicators generally performed well: better than both doc2vec and LDA in predicting mortality and healthcare attendance, albeit with mixed performance in predicting disease occurrence or incidence. This suggests that for many predictive tasks in clinical applications, particularly where model interpretability is important, there is little benefit of bag-of-words methods, particularly given the computational overhead. However, methods such as LDA, unlike doc2vec and transformer methods, may have an advantage in generating interpretable topic distributions which are more easily explainable.^34^ Notably, we found that using the count of all repeated disease codes in the EHR performed particularly poorly and worse than methods using a single code occurrence, which suggests that crude code frequency in the EHR does not relate directly to disease severity and downstream risk. Previous work has highlighted the potential biases in code frequency in primary care data, which may relate to patient sociodemographics, organizational policies, and coding incentives, rather than being an objective marker of a person’s health status.^26^ However, the better performance of BERT models, which also utilize recurrent codes, suggests that an ability of the attention mechanisms in these models to regulate the impact of repeated codes.

The similar performance of embeddings generated from models using diseases (*N* = 212) or clinical codes (*N* = 9462) suggests that the disease categories capture meaningful connections and use of the larger, more granular vocabulary does not add explanatory power. However, the manual grouping of codes into disease categories is a laborious process, that needs to be repeated as old codes are retired and new ones introduced. Our findings suggest that NLP models (as might be expected given their application to the large vocabulary found in natural language) can manage equally well with larger vocabularies and extract relevant latent meaning. By extension, this indicates that inclusion of larger vocabularies of diagnosis codes, or use of other coded information, such as prescription data, may aid in prediction without the need for clinical categorization. The 3 diseases we selected were relatively common; future work could consider whether model inputs result in equal performance at predicting more granular subcategories of diseases, although event rates for these will be low.

Performance also varied according to the task, suggesting that the availability and choice of data inputs for models has a greater impact than the algorithm that generates the embedding. Prediction of any ED attendance was notably poor, which likely relates to absence of other factors relating to attendance in our models, such as proximity and access to both primary care and ED departments and presentations with conditions not strongly related to MLTC, such as injuries.^35^ Although the embeddings generally performed well at predicting any attendance with a disease in the next 12 months, all performed poorly in predicting new diagnoses, with very low APS scores and poor PPVs at different sensitivity thresholds (Figure S6 and Table S8). In the original BEHRT paper (which also used CPRD data), the authors reported slightly higher AUC scores of 0.82, 0.81, and 0.88, compared with 0.76, 0.79, and 0.71 in our study, for hypertension, diabetes, and depression, respectively, which may relate to the additional benefit of fine-tuning beyond the MLM task that we employed.^9^ Solares et al also employed the disease embeddings used by BEHRT as weights to connect the inputs in a neural network, and found relatively higher precision scores (APS 0.15 for detecting hypertension).^8^ This might be explained by their use of a neural network architecture, which will allow for non-linear interactions between features, unlike the logistic classifier used here.

The finding that the more complex transformer representations perform better at predicting a range of clinical endpoints, even without fine-tuning, suggests that in addition to prediction, embeddings could be used for patient clustering or segmentation. These approaches aim to group people based on similar demographic or clinical criteria, for the purposes of risk stratification, such as identifying patients in segments with a high risk of mortality or hospital admission for proactive intervention.^10^^,^^36^ Transformation of data to a vector representation enables patient similarity to be determined for use as a clustering metric; the finding that these vector representations perform better across a range of healthcare outcomes suggests that derived clusters may be more informative with respect to multiple outcomes, and is an promising avenue for future research.

### Strengths and limitations

A strength of our study is the application to a large and representative primary care dataset of 6 million people with MLTC in England.^11^ In contrast to studies using only secondary care data, primary care data contains the longitudinal history of a person’s health from birth to death, making them ideally suited to analyses of disease incidence. However, there are well-recognized biases in routinely collected data which impact on whether a code is recorded.^37^ First, there may be temporal delays in a code being entered, either due to late presentations by a patient, diagnostic uncertainty, or diagnostic delays, waiting until a firm diagnosis or referral is made, or correspondence is received from hospital.^38^ Second, the recurrence of codes in the medical record is dependent on factors including the GP practice, patient demographics, and disease-related factors, such as whether the disease is part of a quality assessment metric for practices.^26^ Previous research has highlighted that those in some ethnic groups, and living in areas of higher socioeconomic deprivation have a greater frequency of recurrent codes, which may result in models having more data, and therefore learning sequences better for some patients than others.^26^ There is also a risk of introducing racial biases when incorporating factors such as ethnicity into clinical algorithms.^39^^,^^40^ Although addition of sociodemographic variables to EHR-BERT improved the predictive performance across the whole cohort, future work could explore whether performance improves for all demographic groups. Furthermore, as with any algorithms used to inform clinical decision-making, careful evaluation would be needed to assess for risk of bias before implementing in healthcare settings.

Our study evaluated clinical outcomes over 1 year up to the end of 2015—prediction of outcomes over longer time periods, for example, 3 or 5 years would likely lead to worse absolute performance of the embeddings. Nevertheless, we would expect relative differences in performance between embeddings to generalize to longer follow-up times. The COVID-19 pandemic has also had a significant impact on population health and health systems. Earlier research has found lower rates of recurrent clinical codes in the EHR associated with the COVID-19 pandemic, although it is unclear whether this relates to changes in patterns of presentation by patients or changes to quality of coding practices by clinicians.^26^^,^^41^ Although we would expect the relative performance of the embeddings to be the same, reductions in the frequency of codes may worsen absolute performance, and should be a focus for future research to understand whether predictive models developed using people’s sequences of diseases before the pandemic perform equally well following the pandemic.

A further strength of our study is the comparison of approaches applied to a single data source. However, a limitation of the large variety of methods trialed is the unavoidable difference in selection of the optimal hyperparameters between algorithms. With LDA, we used the perplexity score to determine the optimal number of topics, whereas for doc2vec, we used a metric based on creating a similar embedding for patients with identical disease sequences. This limitation is inherent to use of unsupervised methods, where there is no ground-truth for evaluating a learned embedding and represented a pragmatic approach. Nevertheless, it is likely that further hyper-parameter optimization could improve upon the learned representations from LDA and doc2vec algorithms. Similarly, and in common with other machine learning approaches, BERT architectures include many hyperparameters which may affect performance. In our work, we used settings identified by other authors, rather than seek to evaluate the optimal configurations for our case, but this represents an avenue for further work. Furthermore, we used patient representations generated from the second-to-last hidden layer; further exploration of the optimal layer or combination of layers may lead to improved performance. However, previous literature applied to language has suggested only small differences between layer choice.^28^^,^^42^ Our final EHR-BERT transformer models ran for 100 epochs, but a sensitivity analysis comparing embeddings at 10 or 50 epochs, showed only minimal degradation of performance, suggesting that computational time could be considerably reduced by running over a smaller number of epochs.

Given our focus on the direct comparison of the embeddings as inputs, we used only logistic regression, given its popularity in epidemiological research, interpretability, and relatively few hyperparameters for tuning. Use of different classification algorithms, such as kernel-based algorithms, multi-layer perceptron or gradient boosting algorithms may result in better predictive performance of the embeddings, particularly given the likelihood of non-linear interactions between features.^6^ However, these algorithms also require a significant amount of optimization, with hyperparameters that are likely to vary both by the embedding input and the outcome, and would complicate direct comparison. Event rates were low for some outcomes, such as new disease diagnoses (Table 3), which may explain the poorer performance and low APS values. While we considered use of under- and over-sampling strategies, given our focus was on relative performance of the embeddings, this might introduce bias to assessment of the best performing embedding. However, it is likely that absolute performance could be improved through sampling methods to address the imbalance in the data.

## Conclusion

Comparing different NLP algorithms for representing patients’ disease patterns, we found that representations generated by algorithms using sequences performed better at predicting clinical endpoints than methods using co-occurrence alone, although with additional computational cost in generating the embeddings compared with baseline approaches. The better performance of unsupervised patient representations from transformer architectures, even without fine-tuning, indicates the potential of these approaches for learning multi-purpose representations which could be used in future for segmentation and risk stratification. Further development of transformer methods for EHRs, and incorporating additional information such as prescription data, holds significant promise for predictive modelling and supporting clinical decision-making in future.

## Supplementary Material

ocae091_Supplementary_Data

## Data Availability

This study uses patient data which is not publicly available but can be requested for users meeting certain requirements: https://cprd.com/research-applications. Code to pre-train EHR-BERT using dummy data, and the Medcode to disease mapping are available from https://tbeaney.github.io/MMclustering/.
